# Reintegration of child soldiers in Burundi: a tracer study

**DOI:** 10.1186/1471-2458-12-905

**Published:** 2012-10-25

**Authors:** Mark JD Jordans, Ivan H Komproe, Wietse A Tol, Aline Ndayisaba, Theodora Nisabwe, Brandon A Kohrt

**Affiliations:** 1 Department of Research & Development, HealthNet TPO, Amsterdam, The Netherlands; 2 Center for Global Mental Health, London School of Hygiene and Tropical Medicine, London, UK; 3 Faculty of Social and Behavioural Sciences, Utrecht University, Utrecht, The Netherlands; 4 Bloomberg School of Public Health, Johns Hopkins, New Haven, USA; 5 Burundi Country Office, HealthNet TPO, Bujumbura, Burundi; 6 Department of Psychology, Bujumbura University, Bujumbura, Burundi; 7 Department of Psychiatry and Behavioral Science, George Washington University, Washington, D.C, USA

**Keywords:** Child soldiers, Reintegration, Mental health, Conflict, Burundi

## Abstract

**Background:**

Substantial attention and resources are aimed at the reintegration of child soldiers, yet rigorous evaluations are rare.

**Methods:**

This tracer study was conducted among former child soldiers (N=452) and never-recruited peers (N=191) who participated in an economic support program in Burundi. Socio-economic outcome indicators were measured retrospectively for the period before receiving support (T1; 2005–06); immediately afterwards (T2; 2006–07); and at present (T3; 2010). Participants also rated present functional impairment and mental health indicators.

**Results:**

Participants reported improvement on all indicators, especially economic opportunity and social integration. At present no difference existed between both groups on any of the outcome indicators. Socio-economic functioning was negatively related with depression- and, health complaints and positively with intervention satisfaction.

**Conclusion:**

The present study demonstrates promising reintegration trajectories of former child soldiers after participating in a support program.

## Background

Where armed conflict exists, child soldiers ^1^[1] will almost certainly be involved. The military recruitment of children and their use in military activities has taken place in one form or another in at least 86 countries and territories worldwide [1]. Even though the past years have seen promising international legal efforts to combat recruitment of children in armed conflicts, the impact of these initiatives still remains insufficient [1]. The plight of former child soldiers has also received significant attention in the academic literature. This is likely due to the assumption that this is a particularly vulnerable population given their traumatic experiences during recruitment and conscription, which is substantiated by several studies [2,3]. Research further demonstrates that the vulnerability of former child soldiers is not limited to the time of conscription; the process of reintegration proves to be an additional stressor for their vulnerability [4-6]. Post-conflict factors such as educational and economic deprivation, stigmatization and discrimination have been associated to adverse outcomes [6-8]. We use the term child soldier to reflect the Paris Principles definition of children associated with armed forces and armed groups, which refers to “any person below 18 years of age who is or who has been recruited or used by an armed force or armed group in any capacity, including but not limited to children, boys, and girls used as fighters, cooks, porters, messengers, spies, or for sexual purposes. It does not only refer to a child who is taking or has taken a direct part in hostilities”[9].

Despite international attention and the earmarking of substantial financial resources for child soldier prevention and reintegration programs, there are surprisingly few systematic assessments of the long term outcomes of such programs and trajectories [10,11]. To the best of our knowledge only two longitudinal reintegration studies have been conducted; a study of life-outcomes of 39 male former child soldiers in Mozambique [12,13], and a study into the risk- and protective factors and mental health among 260 former child soldiers in Sierra Leone [14,15]. Another study, among 658 formerly-recruited girls and other vulnerable young women in Liberia, Uganda, and Sierra Leone, used participatory action research to understand factors contributing to successful reintegration and enable self-help processes [10]. Finally, one cross-sectional study among 1043 ex-combatants in Sierra Leone assessed the determinants of successful reintegration [11]. These studies, combined with other cross-sectional and observational studies, have highlighted post-demobilization factors and interventions that have been associated with successful reintegration, e.g. community sensitization, cleansing rituals, transitional periods in interim care centers, religious support, psychosocial counseling, family mediation and skills & vocational training [13,16,17]. The role of the family in reconnecting children [6,18] and the use of welcoming rituals or traditional healing practices [19] have further been emphasized to aid adaptation and community reconciliation.

The struggles of youth returning from armed conflict are well documented and constitute a threat to their wellbeing. Ugandan women, for example, were met with systematic violence upon family reunification after escaping from the rebel groups [20]. Another study in Uganda found that the failure of reintegration efforts of former child soldiers, was associated with widespread community resistance (due to perceived unaccountability for the crimes committed), ambiguous blending of religious (e.g. notion of repentance) and cultural concepts as *cen* (the revenging spirits of those who have been killed) [21]. On the other hand, several authors have argued that despite the experienced hardship the majority of former child soldiers cope well with their situation [8,13,16,17]. Boothby describes the former child soldiers to have become productive, capable, trusted and caring adults, with better performance on several socio-economic indicators compared to the national average. Manifestations of resilience that are reported include a sense of agency, social connectedness, positive outlook on the future and spirituality among Columbian former child soldiers [22].

The United Nations outlines the goal of disarmament, demobilization and reintegration (DDR) as a process of removing weapons from the hands of combatants, taking the combatants out of military structures and helping them to integrate socially and economically into society, thereby seeking to support ex-combatants so that they can become active participants in the peace process [23]. Reintegration programs for child soldiers typically comprise economic and education support packages such as vocational training, apprenticeships, micro-finance loans, and formal or informal schooling. The intention of these programs is to prevent or mitigate the social, economic, and psychological sequelae of participation in an armed group. There is an urgent need for more knowledge on the long-term reintegration outcomes of former child soldiers and the impact that reintegration programs have in that process.

Burundi, a country in central Africa, has suffered from a 10-year civil war that formally ended with the ceasefire agreement between the government and the last active armed group (Forces Nationales de Libération, FNL) in 2006. Currently, the country is beginning to reap the dividends of the peace process, but it faces formidable challenges in reviving the shattered economy and forging national unity and political instability has remained. Throughout the 10 years of conflict, both Burundian armed forces and insurgent armed political groups recruited and involved children in a variety of capacities. No reliable figures exist on the number of children who have been conscripted, however estimates range between 6,000 and 7,000 [24]. In 2003, after the signing of peace accords with the main rebel parties, prior to the final ceasefire agreement of 2006, Burundi started with the DDR process.

The current study aimed to assess reintegration trajectories several years after demobilization, looking broadly at socioeconomic and mental health indicators of a large group of former child soldiers and never recruited peers, both of whom participated in an economic support program. To the best of our knowledge, it is one of few studies that compare the long-term socio-economic and mental health status between these groups. The retrospective tracer methodology is used frequently in empirical studies among graduates to assess the long-term match between received education and subsequent career paths, also in low and middle income settings [25,26]. The objectives of the present study were two-fold. First, it aimed to compare the present socio-economic and mental health status of former child soldiers and never-recruited peers after participating in an economic support program 4 years prior. Second, the study aimed to assess the role of an economic support program in the perceived reintegration trajectories of both groups through retrospective analyses of socio-economic indicators before receiving support (T1; 2005–06) and immediately afterwards (T2; 2006–07). The study does not, however, evaluate the effectiveness of the program. The present study design is an innovative strategy that was selected in absence of prospective and longitudinal data. In this paper we refer to reintegration as the “process by which children transition into civil society and enter meaningful roles and identities as civilians who are accepted by their families and communities, which is achieved when the political, legal, economic and social conditions needed for children to maintain life, livelihood and dignity have been secured” [9].

## Methods

### Participants and sampling

**Figure 1:**
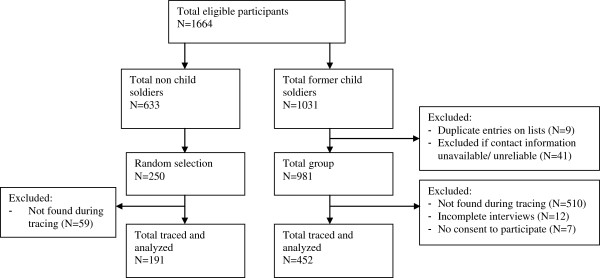
Participant selection Burundi.

All former child soldiers who participated in the reintegration support program (see below) were eligible to be included in this study. In addition, the study also sampled a selection of the non-child soldier beneficiaries who participated in the program (selected based on assessed vulnerability factors such as inability to fulfill basic needs or to attend school) as a comparison group. After finalizing lists of eligible participants (all former child soldiers and a random selection of never recruited children, for whom sufficient tracing information was available) (see Figure 1), actual tracing was commenced to locate target respondents and subsequently conduct interviews. Different strategies were used for tracing: (a) organizations, associations and local authorities were contacted to locate persons; (b) one person was hired to contact people on the list prior to the actual interviews through home visits; (c) snowball sampling once former beneficiaries were traced, which entailed that contacted respondents were subsequently asked to locate other former beneficiaries. We were able to trace and interview 452 former child soldiers and 191 never recruited children. Tracing and subsequent data-collection was done in the period April – August 2010.

### Reintegration support program

The International Labor Organization (ILO) conducted a program that aimed to benefit the (economic) reintegration into civilian life of former child soldiers and prevent future recruitment of at-risk children that have not been recruited (the comparison group as described above). Providing the support program to both groups was done for reasons of equity, non-stigmatization and the promotion of integration [9,27]. The intervention consisted of one or several components following ILO’s strategy for the economic reintegration of former child soldiers: (a) training for economic empowerment, including short and long vocational training, entrepreneurship training (i.e. basic management and accountancy courses), on-the-job training, informal education and/or life-skills training, and (b) assistance in starting and maintenance of self-employment, including coaching and support in the management and formalization of small business associations, equipment kits or start-up materials and/or micro-finance support (i.e. increasing financial literacy, help in opening a bank account, encouraging savings, access to and management of micro-credit schemes). While no standard method of implementation was followed, there were several commonalities in approach. All implementing partners formed associations, defined as small cooperatives of persons united voluntarily to meet common economic needs through joint and democratically-controlled micro-enterprises. In addition the programs included an awareness raising component, targeting negative community attitudes towards former child soldiers. Special attention was given to gender equity, both in terms of promoting the needs and contributions of young women, as well as in fine-tuning activities to their aspirations. Vocational training and micro-enterprises included pork and goat rearing, soap making, beekeeping, sewing, woodwork and car-mechanics. These economic empowerment services, it was conceived, would help to increase the productivity, self-sufficiency and normalization of former child soldiers, aiding their readjustment to civilian and community life [27]. Subsequently, initial post-intervention improvements in socio-economic integration were expected to have a long-term impact on functioning and psychosocial well-being.

### Procedure

We conducted a tracer study based on a methodology to measure retrospectively long term outcomes for children who have been involved in the ‘worst forms of child labour’ [28]. ILO Convention 182 prohibits the worst forms of child labour and defines the ‘forced or compulsory recruitment’ of children below 18 for use in armed conflict as one of these worst forms. We used the tracer methodology to retrospectively document former beneficiaries’ perspectives on selected socio-economic outcome indicators for the period directly after demobilization and before participating in the support program (T1, between 2005 and 2006), the period directly afterwards (T2, between 2006 and 2007), and the present moment (T3, 2010). The scores for the periods T1 and T2 are recollections, while T3 data present the cross-sectional characteristics of the sample population. In other words, data was only collected one time, i.e. during the 2010 interviews. In a situation where baseline information of social, economic, and education status is missing, tracer studies are used to estimate changes over time.

Informed written consent was taken prior to starting the interview and confidentiality was assured by explaining procedures of data storage and anonymity. Approval for the study was gained from local authorities (written authorization from the provincial Governor, and subsequently permission from *Chef de Colline*, administrator of the smallest administrative unit in Burundi, where data collection took place) and data collection procedures were consistent with the Declaration of Helsinki [29]. Approval was granted to the authors for conducting the interviews in 2010, no other data was collected. We followed regulations for ethical considerations for conducting tracer studies as stipulated by ILO, who commissioned the study. There was no ethics committee in Burundi to review this study. The research was part of a broad nation-wide service delivery program for at-risk populations carried out by local staff and organization. Interviewers with existing research experience were retrained for a 5-day period. The training included the following topics; basic research skills, research ethics, communication and interviewing skills, field-testing of the instrument, data entry, logistics, harm to self and referrals of respondents identified with serious mental health due to this research.

### Instruments

The interview consisted self-report questionnaires, covering the following primary outcome variables: (a) work satisfaction (at T1, T2 and T3); (b) household economic well-being (at T1, T2 and T3); (c) social integration (at T1, T2 and T3); (d) perceived economic opportunities (at T1, T2 and T3); as well as socio-demographic information. Secondary outcome indicators were included to assess current level of function impairment and symptoms of depression, anxiety and posttraumatic stress disorder (PTSD) (at T3). Assessment of current mental health was included as a proxy-indicator of functioning or adjustment, especially given the described association between child soldiering and mental health problems. Details of child soldier experiences were collected, including the type of experiences during association with the armed group. Finally, participants provided details of the type of, and satisfaction with, program support received, with the latter referred to as program endorsement.

Several of the instruments were constructed for the purpose of the study (ILO, 2010b). The scale ‘*social integration*’ consisted of 7 items with a 4 point response format to assess the level of perceived acceptance by peers, family and community (α=.85; e.g. How much did you feel accepted by the community? How much did you participate in community activities? Did you feel cared for by your family? How did your life compare to other persons of your age from the community?). The scale ‘*work satisfaction*’ consisted of 4 items with a 4 point response format to assess the level of satisfaction with conditions of labor (α=.63; e.g. Was the income you made sufficient to sustain yourself and family? How would you rate your work conditions?). The scale ‘*household economic wellbeing*’ consisted of 8 items with a 3 point response format to assess the level of satisfaction with the family’s livelihood situation (α=.82; e.g. Did your household own animals? Was your household able to pay for medical expenses? Did your family have sufficient food to eat [3x/day]?). The scale ‘*economic opportunities*’ consisted of 4 items with a 4 point response format to assess the level of perceived economic prospects (α=.80; e.g. How did you perceive your future? How did your economic opportunities compare to those of your peers? Do you think you can improve your economic situation? ). The scale ‘*program endorsement*’ consisted of 5 items with a 4 point response format to assess the level of satisfaction with the support program the respondents participated in (internal consistency α=.84; e.g. Were you satisfied with the support you received? Did the received support meet your needs?). The scale to measure *impairment in functioning* was developed by Bolton and colleagues [30] for a study in neighboring Rwanda. The tool consisted of 7 items for male respondents (α=.75) and 9 items for female respondents (α=.83) with a 5 point response format (higher scores indicate higher levels of impairment). Standard instruments were used to assess symptoms of depression, anxiety and PTSD. We used the Hopkins Symptom Checklist (HSCL-25) to screen for symptoms of *depression* (α=.83; international cut-off score for caseness > 1.75) and symptoms of *anxiety* (α=.81; cut-off score > 1.75) [31]. To assess symptoms of *Post Traumatic Stress Disorder* we used the PTSD Checklist (PCL-c) (α=.90; cut-off score > 50) [32]. Instruments were translated using a method developed for transcultural research [33,34].

### Analyses

We ran descriptive statistics for current levels of socio-economic functioning, function impairment and mental health symptoms and disorders. Caseness for each of the assessed disorders was determined by identifying respondents scoring above cut-off scores on symptom checklists combined with moderate impairment in functioning (respondents that have moderate impairment in functioning on at least one of the daily activities and some impairment in functioning on others) [35]. Subsequently, to test differences between former child soldiers and never recruited respondents, we conducted χ^2^ tests (with Fisher exact test) for comparison of categorical data and independent sample t-tests for between-group comparison of means of continuous variables.

To explore associations between the outcome variables and a set of predictor variables (i.e. demographics, child soldier experiences, received program support, mental health), we followed a 3-step procedure (cf. [36]). The predictor variables were selected based on the premise that they influence the reintegration trajectories of former child soldiers. First, we calculated a Pearson correlation matrix from the predictor and outcome variables. Second, the predictor variables that were significantly correlated (*p* < 0.05) with one of the study outcome variables were entered as blocks in separate hierarchical regression analyses. Block 1, demographics: gender, age, marital status, educational status; Block 2, child soldier experiences: activities during association, reasons for association, duration of association, rejoined armed groups, combat experience; Block 3, (mental) health symptoms: anxiety, PTSD, depression, function impairment, health complaints; Block 4, program components: vocational training, awareness raising, non-formal education, job-coaching, life skills training, formal education, internship, entrepreneur training, micro-financing, material kits, length of intervention, intervention satisfaction. Third, all predictor variables with significant β-coefficients found at the previous step were entered in one multivariate regression analysis per study outcome variable. For each outcome variable we estimated adjusted R squares (δR^2^) and betas (β) for each predictor variable. Statistical significance was defined as p < 0.05.

Finally, for analyses of changes in means between the three time points, pure change scores for the outcome measures (social integration, household economic well-being, perceived economic opportunity, work satisfaction) were calculated, which were compared with paired sample t-tests. We used MANOVAs to test for an effect of time for the entire group and for a group x time interaction effect to assess differences between the two groups.

## Results

The sample (N=643) consisted of 470 boys (73.1%) and 173 girls (26.9%), aged between 14 and 30 years (mean 22.54; SD 3.51), of whom 267 (43.3%) were presently married. Six hundred thirteen (95.3%) respondents were Christian, 16 Muslim (2.5%) and 14 reported no religious affiliation (2.2%). Table 1 shows demographic information, as well as recalled baseline scores on outcome measures, separately for former child soldiers and never recruited respondents. There were significant differences between both groups for gender, age, literacy and baseline level of social integration, with former child soldiers demonstrating lower perceived social integration. Former child soldiers joined the armed groups at an average age of 14.6 years old (SD = 3.02) and stayed associated for an average of 4.2 years (SD = 2.45). The majority of them reported to have made the decision to join the armed groups voluntarily (69.4%) followed by forced recruitment or abduction (16.3% and 8.4%, respectively). The reasons for ‘voluntary’ recruitment were mainly to gain material benefits (32.6%), out of fear (16.2%), the prestige of being associated with armed groups (13.5), peer influences (13.0%), ideology (10.7%), desire for vengeance (7.3%) and a sense of being socially excluded (6.6%). Once recruited the activities that the former child soldiers were asked to carry out included combat experience (50.7%), being a porter (58.4%), cooking food (40.7%), being a sentry (11.7%), spying (12.6%) or delivering messages (2.0%). Total percentages exceed 100% as each person could respond with multiple answers to the above items.

**Table 1:** Sample characteristics

**Characteristic**	**Former child soldiers (N=452)**	**Never recruited (N=191)**	***p *value**
Male, N (%)	391 (86.5%)	79 (41.4%)	.000
Age ^a^, mean (SD)	23.53 (3.19)	20.31 (3.16)	.000
Household members, mean (SD)	5.02 (2.32)	5.40 (2.36)	.066
Literacy, N (%)	391 (87.1%)	152 (80.0%)	.031
Outcome baseline indicators ^b^, mean (SD)			
Social integration	14.80 (4.15)	18.71 (2.86)	.000
Economic opportunities	7.83 (2.94)	8.07 (3.12)	.366
Household wellbeing	12.83 (3.34)	13.23 (3.61)	.187
Work satisfaction	11.50 (2.45)	11.60 (2.52)	.692
Program endorsement, mean (SD)	14.77 (3.99)	15.44 (3.69)	.057

Note: ^a^ Age at the time of the interview; ^b^ On all outcome indicators a higher score denotes a more favorable situation.

We wanted to assess present socio-economic functioning and mental health of former child soldiers as proxy-outcome indicators of long-term reintegration, and compare that to never-recruited peers. The level of impairment in daily functioning among the child soldiers group was rather low with a mean score of 9.99 (SD=3.86), considering the 7 to 35 response scale. With regards to mental health problems, we saw prevalence rates of 13.3% depressive disorder, 14.3% anxiety disorder and 5.9% PTSD, based on the algorithm that combines above cut-off symptoms scores with function impairment scores. Table 2 further shows that there were no significant differences for present functioning impairment or mental health problems between former child soldiers and never recruited respondents. However, there was a gender difference: former girl child soldiers have significantly more depression (t(161)=2.18; p=.043) and PTSD (t(154)=2.04; p=.031) symptoms at present compared to never recruited girls.

**Table 2:** Present levels of mental health symptoms and functioning impairment

**Characteristic**	**Total group**	**Caseness**	**Former child soldiers (N=452)**	**Never recruited (N=191)**		**Boys**	**Girls**
	Mean (SD)	%	Mean (SD)	Mean (SD)	*p* value	*p-*value	*p-*value
Depressive symptoms	27.72 (7.31)	13.3	27.87 (7.34)	27.37 (7.23)	.441	.461	.043
Anxiety symptoms	20.85 (5.65)	14.3	20.82 (5.69)	20.91 (5.58)	.855	.098	.255
PTSD symptoms	40.00 (12.42)	5.9	40.43 (12.60)	39.03 (11.99)	.208	.603	.031
Function impairment	9.99 (3.86)	n/a	10.13 (3.68)	9.64 (4.30)	.144	.850	.605

Note: PTSD = Post Traumatic Stress Disorder.

Hierarchical regression analyses demonstrated predictors for present socio-economic functioning of former child soldiers (see Table 3). When adjusting for mutual effects of the different blocks, we saw that older age, not having a partner, lower functioning impairment, lower levels of depression- and health problems, received material kits and higher intervention satisfaction are associated with increased household wellbeing. Participating in awareness raising programs is negatively associated with household wellbeing. Increased social integration is associated with less depression symptoms, fewer activities conducted during associations, received vocational training and on-the-job training, and higher intervention satisfaction. Present levels of perceived economic opportunities are associated with higher number of conducted activities during association, less depression- and health- problems and higher intervention satisfaction. Finally, more activities conducted during association, less depression- and health- problems, received job-coaching and higher intervention satisfaction are significantly associated with higher present work satisfaction. At present 94.6% of the former child soldiers mention to have some sort of employment or to be working and 87.1% report to be literate.

**Table 3:** Predictors for socio-economic indicators (Burundi)

	**Household economic wellbeing**		**Social integration**		**Economic opportunity**		**Work satisfaction**	
	***β***_**1**_	***β***_**2**_	***β***_**1**_	***β***_2_	***β***_**1**_	***β***_**2**_	***β***_**1**_	***β***_**2**_
		**δ*R***^**2**^_**2**_=.34**		**δ*R***^**2**^_**2**_=.25**		**δ*R***^**2**^_**2**_=.40**		**δ*R***^**2**^_**2**_=.29**
Demographics	δ*R*^2^_1_ =.01*		δ*R*^2^_1_=.00		δ*R*^2^_1_=.03**		δ*R*^2^_1_=.01	
Age	.12*	.11*	-.03		.08		.10	
Gender [male=1; female=2]	<.00		.01		-.12**	-.04	-.05	
Marital status [no partner=0; partner=1]	-.15**	-.18**	.08		-.04		-.04	
Child soldier experience	δ*R*^2^_1_=.01		δ*R*^2^_1_=.05**		δ*R*^2^_1_=.01		δ*R*^2^_1_=.01	
Number of activities during association	.09*	.07	-.23**	-.13**	.13**	.15**	.12*	.11**
Rejoined armed grou ps	.08		.06		.02		.02	
Duration of association	.04		-.02		<.00		<.00	
(Mental) health and functioning	δ*R*^2^_1_=.31**		δ*R*^2^_1_=.20**		δ*R*^2^_1_=.34**		δ*R*^2^_1_=.28**	
Anxiety symptoms	-.04		.04		.04		-.03	
Depression symptoms	-.23**	-.29*	-.47**	-.41**	-.36**	-.36**	-.27**	-.25**
PTSD symptoms	-.08		.06		-.02		.04	
Functioning impairment	-.11**	-.10*	-.08		-.02		-.03	
Health problems	-.29**	-.26**	-.04		-.36**	-.30**	-.36**	-.32**
Intervention received	δ*R*^2^_1_=.08**		δ*R*^2^_1_=.08**		δ*R*^2^_1_=.09**		δ*R*^2^_1_=.07**	
Intervention type								
Awareness	-.10*	-.12**	.05					
Coaching			.10*	.02			.10*	.10*
Vocational training	-.06		.11*	.11**				
Material kits	.12**	.11**						
Basic education	-.08		.03					
On-the-job training			.10*	.12**			.07	
Microfinance			.04				<.00	
Intervention satisfaction	.21**	.15**	.13**	.10**	.30**	.22**	.21**	.15**

Note. * *p*<.05; ** *p*<.01; *β*_1_ = estimates from variables adjusted for the co-variances with other variables in their clusters**;***β*_2_ = estimates from significant cluster variables adjusted for the covariance with other variables from other clusters.

To better understand the above-mentioned findings, i.e. non-difference between groups on several indicators of current functioning and the role of participation in the support program, we have assessed and compared perceived change trajectories of both groups through retrospective assessment of socio-economic indicators before participating in the support and immediately afterwards. MANOVA analyses demonstrated significant changes over time reported program by the entire sample (see Table 4). Comparisons of mean scores between the different time points showed that the largest changes on indicators of socio-economic reintegration were seen between pre- and post program participation, statistically significant for each of the outcome measures. Between T2 and T3, we saw further minor improvements for social integration and household wellbeing (3 to 5%), no significant change for work satisfaction and a small reduction in perceived economic opportunity. Changes on mean scores were the largest for social integration and perceived economic opportunity (21 and 47% pre- post change, respectively).

**Table 4:** Within-group comparisons of mean scores (N=643)

	**Pre score (T1)**	**Post score (T2)**	**Follow-up (T3)**	**Comparison T1-T2**		**Comparison T1-T3**		**Comparison T2-T3**		**Time effect**	**Time effect (boys)**	**Time effect (girls)**
Indicator	Mean (SD)	Mean (SD)	Mean (SD)	% Change	t-value; *p*	% Change	t-value; *p*	% Change	t-value; *p*	F(df); *p*	F(df); *p*	F(df); *p*
Social integration	15.97 (4.21)	19.40 (2.61)	20.29 (2.42)	21.48	25.70; <.000	27.05	26.72; <.000	4.59	10.44; <.000	F(2,1204)= 409.66; <.000	F(2, 872)= 182.56; <.000	F(2, 328)= 163.63; <.000
Economic opportunities	7.90 (3.00)	11.64 (2.86)	11.38 (2.81)	47.34	25.38; <.000	44.05	23.36; <.000	-2.23	-2.78; .001	F(2, 1070)= 322.35; <.000	F(2, 780)= 176.69; <.000	F(2, 286)= 100.83; <. 000
Household wellbeing	12.96 (3.42)	14.31 (3.43)	14.75 (3.27)	10.42	14.68; <.000	13.81	15.22; <.000	3.07	4.49; <.000	F(2, 1138)= 136.46; <.000	F(2, 824)= 72.74; <.000	F(2, 310)= 35.33; <.000
Work satisfaction	11.53 (2.47)	12.10 (2.27)	12.20 (2.19)	4.94	5.63; <.000	5.81	6.76; <.000	.83	1.17; .245	F(2,764)= 21.41; <.000	F(2, 566)= 18.96; <.000	F(2, 194)= 3.23; .042

*Note.* T1 represents “pre-intervention”, T2 represents “post-intervention” and T3 represents current study period (April-August 2010).

Table 5 reports pure change scores and MANOVA results to illustrate comparisons of changes between former child soldiers and never recruited respondents. These analyses showed significant time x group interaction effect for social integration (F(2,1204)=113.18; p<.001) and economic opportunity (F(2,1070)=3.26; p=.039). Former child soldiers reported significantly more improvement on social integration, to the point that baseline differences between both groups were statistically not significantly different at present (t(df)=−.52(628); p=.604). The interaction effect for perceived economic opportunity is explained by a reduction in mean score between T2 and T3 among the never recruited respondents, compared to no change among former child soldiers.

**Table 5:** Between-group comparisons of mean changes

	**Comparison between T1 and T2**		**Comparison between T1 and T3**		**Comparison between T2 and T3**				
	**Former child soldiers**	**Never recruited**	**Former child soldiers**	**Never recruited**	**Former child soldiers**	**Never recruited**	Time * Group effect	Time * Group effect (boys)	Time * Group effect (girls)
Indicator	Change, Mean (SD)	Change, Mean (SD)	Change, Mean (SD)	Change, Mean (SD)	Change, Mean (SD)	Change, Mean (SD)	F(df); *p*	F(df); *p*	F(df); *p*
Social integration	4.32 (3.36)	1.36 (2.02)	5.45 (4.02)	1.71 (2.54)	1.12 (2.17)	.32 (1.77)	F(2, 1204)= 113.18; <.000	F(2, 872)= 182.56; <.000	F(2, 328)= 51.89; <.000
Economic opportunities	3.88 (3.56)	3.63 (3.60)	3.68 (3.50)	2.82 (3.46)	-.17 (2.74)	-.79 (3.57)	F(2, 1070)= 3.26; .039	F(2, 780)= .83; .438	F(2, 286)= 25.11; .019
Household wellbeing	1.44 (2.34	1.28 (2.22)	1.86 (2.88)	1.77 (3.04)	.44 (2.16)	.46 (2.52)	F(2, 1138)= .19; .826	F(2, 824)= .28; .755	F(2, 310)= .70; .819
Work satisfaction	.65 (2.55)	.74 (2.29)	.94 (2.55)	.57 (2.34)	.21 (2.23)	-.10 (2.32)	F(2,764)=1.98; .139	F(2, 566)= .37; .689	F(2, 194)= .90; .408

*Note.* T1 represents “pre-intervention”, T2 represents “post-intervention” and T3 represents current study period (April-August 2010).

Considering the large gender differences between former child soldiers and never recruited respondents, we ran analyses separately for boys and girls. The time effect remained significant for all outcome measures when comparisons were made for only girls and only boys. When comparing the time x group effect for both genders separately, we found that the significant interaction effect for perceived economic opportunities is present only among girls. This means that the smaller overall improvement in economic opportunity (or slight reduction between T2 and T3) among never recruited respondents is explained by change of the girls within this group.

## Discussion

The present study supports the notion of the long-term resilience – when provided with support in socio-economic reintegration - of former child soldiers, as was found earlier in a smaller study with Mozambiquan child soldiers [12]. Overall, the findings illustrate that former child soldiers in Burundi feel by and large socially integrated within communities, with high work/employment rates, literacy rates above national average (66%) and no differences in present functioning and mental health compared to never-recruited peers. This perspective of resilience is further supported by several trends.

First, several years after demobilization the majority of the former child soldiers appear to function no different than their non-recruited peers. The two groups are surprisingly similar in current socio-economic functioning and mental health status. We hypothesize that post-program improvements (most notably in social integration) had a buffering effect for current mental health problems, which contributed to the non-difference between both groups at present. This will need to be confirmed in a future study, but this is congruent with the findings by Betancourt and colleagues that with increased community acceptance youth showed significant improvements in all outcomes investigated [14,15]. Child soldier status and experiences (including combat) explained very little variation in present functioning, with the number of activities performed during recruitment the only recurring predictor. This compares to data from other studies that demonstrate a strong negative association between child soldiering and functional status [3,14,15]. The cross-sectional study of Kohrt and colleagues [3], for example, demonstrated that former child soldiers displayed greater severity of mental health problems compared to never conscripted children. It appears that in time, and after participating in a support program, the effects of soldiering fade to an extent, and more current concerns and stressors take the overhand (i.e. poverty, unemployment), much alike the general population.

Second, socio-economic reintegration trajectories showed significant improvements over time. While this trend was present for each of the indicators, it was especially salient for perceived economic opportunity and social integration. The positive trend in economic opportunity can in our opinion best be explained by former child soldiers’ engagement in, or hope for, new occupational activities or skills (as a result of the support program), and the trend in social reintegration by the normalizing and equalizing effect of performing occupational activities, much alike everyone else, and the associated sense of recognition and utility within their respective communities. Taking into account that lost economic opportunity was considered one of the most devastating legacies of recruitment and that social or community acceptance is considered a major indicator for successful reintegration [10,13], these are salient findings. The findings in the present study are congruent with a key conclusion in the study by McKay and colleagues, that “to garner one’s own resources to move them from being marginalized young mothers to contributing and respected members of their communities was considered true integration” [10]. Similarly, community connection and a sense of future were also identified as protective factors among Columbian former child soldiers in the study by Cortes and Buchanan [22]. In addition, the improvement in social acceptance is important in light of studies demonstrating the centrality of stigma and discrimination in predicting post-conflict adjustment and well-being [6,37]. Changes in household economic wellbeing and work satisfaction were much more modest, mostly due to the pervasive unemployment and poverty in Burundi.

The observed reintegration trajectories suggest a positive role of the support program, given that practically all reported change occurred between pre and post participation. In addition to change trajectories, hierarchical regression analyses also point towards the positive effects of the reintegration program. In comparison, Betancourt and colleagues [14] found no significant changes in levels of community acceptance – in the absence of an intervention - two years after baseline measurements. While coaching, on-the-job and vocational training and provision of material kits were associated with improved socio-economic reintegration trajectories, a sense of satisfaction with received services is most strongly and recurrently associated with better outcomes.

Third, we see modest signs towards positive gain for former child soldiers. They outperform, albeit slightly, their never recruited peers in perceived economic opportunity. The activities performed during association appear to provide a sense of empowerment. The respondents reported to have gained skills and experiences that they could use post-demobilization. In a study among former child soldiers in Nepal, a similar trend was detected [38]. The authors posit the concept of ‘unbalanced agency’ to refer to the discrepancy between the benefits that children gain through participation in armed groups and the obvious risks associated with it.

Fourth, the present levels of mental health complaints and impairment in daily functioning is not higher than rates found among the general population in other studies in similar post-conflict and neighboring settings (i.e. Rwanda, Uganda) [30,35]. Still, the presented (mental) health complaints call for serious attention, especially depression and health problems since they are most strongly associated with reduced current socio-economic functioning, more so than gender or previous experiences during association with armed groups. This is something that should be explored in future studies.

The current study finds that girls are more at risk to suffer from depression and PTSD complaints, a replication of the findings by Kohrt and colleagues [3] directly after demobilization, but otherwise show no difference in the reintegration trends compared to boys. The latter is surprising as previous studies have demonstrated former girl soldiers to be more vulnerable for experiencing reintegration difficulties like stigma [6] and functional complaints [4]. With many girls falling through the cracks of the reintegration services in other DDR systems [1,17], it may be that the reintegration process in Burundi was more gender-equitable, especially since this was emphasized in ILO’s economic reintegration strategy [39]. Additionally, Humphrey and Weinstein in their large survey also conclude that gender is not a predictor for increased difficulties for reintegration into civilian life [11]. Furthermore, another study in Northern Uganda demonstrated that most women returning from armed groups were resilient and well reintegrated socially [40].

The most important limitation to this study is related to the retrospective nature of the design. All scores are based on recollections, which is sensitive to introducing recall bias. Regarding the retrospective design, those with high levels of current mental health problems and psychosocial distress are more likely to appraise earlier experiences negatively and recall stressful life events, and they are less likely to recall experiences of support [41,42]. Second, the lack of a control group makes it harder to determine causality of the socio-economic improvements. With a control group, there would be greater ability to assess what changes in mental health, social indicators, and economic activity would have occurred in the absence of any formal intervention. For example, it is conceivable that the reported changes are due to factors other than the support program, such as increased security, passage of time, increased reconciliation or other humanitarian efforts. Yet, given that most of the change occurred in the short time span between pre- and post program participation suggests that it had an impact on the reported changes. This is also confirmed by the results from the hierarchical regression analyses. The findings of this paper should be interpreted with caution. Future research should include a control group and a longitudinal design with a baseline measure to facilitate attribution of changes over time to reintegration activities.

Another limitation is that the standard symptoms checklists, while demonstrating good internal consistency, were not assessed for construct or concurrent validity within the Burundian context. The qualitative approach used does address transcultural equivalence of the tools. However, a validation study is required to determine to what degree the instruments distinguish between individuals with and without a particular syndrome or disorder [33,34]. This may have had an impact on the proportion of caseness within the sample. To adjust for the common risk of inflated prevalence when using non-validated instruments, we have determined caseness based on a combination of symptoms levels and function impairment, which is a technique used in other cross-cultural studies [30]. To address the limitation of generalizability due to un-validated instruments, further research is necessary to assess construct validity of the measures for this specific population. Finally, the use of non-random sampling procedures for the interviews may limit the representativeness of the sample. The present study included only beneficiaries of integration support, yet many other former child soldiers do not receive any such support [1], their long-term wellbeing should be assessed in future studies.

This study has several implications for policy and practice. Policy makers and program planners may interpret these findings as heartening. Long-term socio-economic and mental health outcomes are not different between former child soldiers and never recruited children, with participation in an economic support program appearing to contribute to perceived improvements over time. Based on the finding that the association between outcomes and program satisfaction is the strongest in the tested model, one could argue for increased participation in designing reintegration services to augment the match between needs and services and build on their positive coping (and developed skills) as a result of being recruited, in order to increase satisfaction. Such shift has also been advocated by others [8,10,17]. It should be noted that a reverse causality of this finding is also possible, i.e. individuals with more positive outcomes are most likely to be satisfied by the program. Second, future (economic) reintegration packages should emphasize apprenticeship activities (e.g. vocational- and on-the-job training, coaching). Third, while it poses inherent limitations, retrospective tracer studies may offer a useful method for the assessment of long term outcomes and an alternative to prospective longitudinal studies in absence of pre-planned studies. For researchers interested in studying complex interventions in low-resource settings it provides for an additional tool to strengthen the evidence base and elucidate processes to be included in future more rigorous efficacy studies.

Finally, we would argue that successful reintegration is about the *equality* in opportunities, participation, wellbeing and social functioning of former child soldiers compared to those of their never-conscripted peers. Whether or not a former child soldier has found employment, is married, received education or experiences few mental health problems, are indicators of long-term reintegration primarily vis-à-vis the average of the population at large or their peers. From such a perspective the results of this study are hopeful, but no more hopeful than the general situation of Burundi, with a population that is faced with significant socio-economic adversities, structural marginalization and continued community violence. The results do not diminish the undergone hardship or invasive consequences of the respondents’ experiences, but demonstrate that despite these, and with the support from reintegration services, former child soldiers have integrated seemingly well.

## Conclusion

The reintegration trajectories of former child soldiers are largely positive, demonstrating considerable resilience. Present day functioning is no worse than that of their never recruited counter-parts. A reintegration support program appears to have had played a positive role in improved socio-economic well-being. Such effect was mainly observed on psychosocial, rather than economic indicators and was best predicted by a sense of satisfaction with received services. Current levels of mental health problems among former child soldiers are not alarming when compared to the general population in the region. The results of this study, combined with other studies into the reintegration of former child soldiers, are cautiously positive and, despite their tragic experience, provide hope for the long-term wellbeing of this group.

## Competing interests

The authors declare that they have no competing interests.

## Authors' contributions

MJ designed and supervised the study, conducted analyses and drafted the manuscript. IK has been involved in study design, statistical analyses and revising manuscript. WT has been involved in study design and revising manuscript. AN has been involved in the design of the study, acquisition of the data and revising of the manuscript. TN has been involved in the design of the study, overseeing data acquisition and revising of the manuscript. BK has been involved in study design, statistical analyses and revising manuscript. All authors read and approved the final manuscript.

## Endnote

^1^ We will use the term ‘child soldiers’ throughout the paper to refer to the broader term ‘children formerly associated with armed forces and armed groups (CAAFAG)’, for reasons of readability.

## Pre-publication history

The pre-publication history for this paper can be accessed here:

http://www.biomedcentral.com/1471-2458/12/905/prepub
